# Effects of NBP on postoperative cognitive dysfunction in rats via Nrf 2/ARE pathway

**DOI:** 10.18632/aging.204481

**Published:** 2023-01-09

**Authors:** Jianshuai He, Junqiong Gao, He Zhu, Yang Zhao, Xiaotian Zhang, Xiufang Wang, Shengnan Wan, Hongying Cao, Lin Zhai, Yuanyong Wang, Shilei Wang

**Affiliations:** 1 Department of Anesthesiology, The Affiliated Hospital of Qingdao University, Qingdao, China; 2 Department of Anesthesiology, Weihai Municipal Hospital, Weihai, China; 3 Department of Thyroid Surgery, The Affiliated Hospital of Qingdao University, Qingdao, China; 4 Department of Thoracic Surgery, Tangdu Hospital of Air Force Military Medical University, Xi’an, China

**Keywords:** NBP, POCD, Nrf 2/ARE pathway, anesthesiology, rat

## Abstract

Objective: Postoperative cognitive dysfunction (POCD) is a common postoperative disease that threatens patients’ quality of life, especially elderly patients. With the popularity of anesthesia/surgery, POCD has received more attention worldwide. The objective of this research is to evaluate 3-n-Butylphthalide (NBP)’s protective effect on postoperative cognitive function in rats and its related mechanisms.

Methods: Tibial fracture models of senile rats of POCD were established and divided into blank control group, solvent group, NBP group, Nrf 2 agonist group, and Nrf 2 inhibitor group. The changes in the cognitive abilities of rats were systematically evaluated by the Morris water maze test. After hematoxylin-eosin (HE) staining of the hippocampus, the morphological and structural changes of hippocampal neurons were observed by light microscopy. The expressions of apoptosis-related proteins were analyzed by immunohistochemistry and Western blot was used to detect the expressions of Nrf 2,HO-1,Mfn1,Mfn2,Drp1 proteins. Moreover, the changes in the morphology of mitochondria were observed by transmission electron microscopy.

Results: Through the water maze test, we observed that the incidence of postoperative cognitive impairment in the NBP, agonist, and inhibitor groups was substantially lower as compared to the blank control group and solvent group (P < 0.05). The expressions of Nrf 2, HO-1, Mfn1, Mfn2, and Drp1 proteins in the NBP group were upregulated in comparison to the blank control group and the solvent group. The expressions of related proteins in the inhibitor group were substantially lower in comparison to the NBP group.

Conclusions: NBP can affect the postoperative cognitive function of rats by activating the Nrf 2/ARE signaling pathway.

## INTRODUCTION

Postoperative cognitive dysfunction (POCD) is a common central nervous system (CNS) complication after waking up from surgical anesthesia, which is characterized by the decline of memory, attention, learning ability, information processing ability, and social ability [1, 2]. The occurrence of POCD is high in the elderly over 65 years old [3]. Moreover, POCD not only increases the length of hospital stay and hospital costs but also increases the incidence of postoperative complications and mortality rate [4]. With the progress of medical technology and the arrival of an aging population in China, the number of elderly patients undergoing surgery will gradually increase. As a result, the number of POCD patients will also increase, and it will cause an enormous economic strain on the country and its families [5]. Therefore, the study of POCD has been the key research direction of anesthesiology, neuroscience, and geriatrics in recent years.

At present, most of the studies on the Nrf 2/ARE pathway in neurocognitive disorders focus on the downstream products of the Nrf 2/ARE pathway and their effects on oxidative stress and neuronal apoptosis, lacking studies on the direct impact of Nrf 2 on mitochondrial function [6]. Currently, most of the studies are focused on vascular cognitive impairment (VCI) and Alzheimer's disease (AD), and only a few studies are on POCD [5, 6]. Therefore, we conducted this study to investigate the function of the Nrf 2/ARE signaling pathway in POCD through the rat tibial fracture model of POCD and verify the protective effect of the Nrf 2/ARE signaling pathway on POCD through the specific agonists and antagonists of this pathway from the aspects of rat behavior test, hippocampal neuron morphology, hippocampal tissue oxidative stress level, and mitochondrial morphology and dynamics in hippocampal neuronal cells.

NBP is the latest National Class I drug synthesized by our country, which is widely used in the treatment of stroke and vascular dementia and has good clinical efficacy [7]. Animal experiments have confirmed that NBP has an antioxidant effect by activating the Nrf 2/ARE pathway in rats with cerebral ischemia-reperfusion injury [8]. It has not been confirmed whether NBP affects mitochondrial division in nerve cells. Currently, most of the studies on butylphthalide are focused on stroke and vascular dementia, but there is no study on its use in POCD. Therefore, we designed this experiment to observe the effect of butylphthalide in the animal model of POCD and explore its clinical application value through clinical trials.

## MATERIALS AND METHODS

### Experimental animals

Thirty healthy Sprague dawley (SD) rats of Specific pathogen Free (SPF) grade, aged 6 to 7 months, weighing 350 to 420 g, were purchased from Jinan Pengyue Experimental Animals Breeding Co., Ltd. (China) and maintained under a natural light source at a temperature of 23 ± 1° C. They were provided access to unlimited water and food, and the experiment was carried out one week after the adaptive feeding.

### Reagent and apparatus

The reagents and apparatus were purchased from the following companies: the NBP (Catalog No. HY-B0647, 200 mg/tube), Tert-butylhydroquinone (TBHQ) (Catalog No. HY-100489, 500 mg/tube), Brusatol (Bru) (Catalog No. HY 19543, 10 mg/tube), PEG 300 (Catalog No. HY-Y0873, 100 ml/vial) and HO-1 antibody (HY-P70276) were purchased from MedChemExpress Co. Ltd.; caspase-3 antibody (4.1.18) (Catalog No. SC-65497), caspase-9 antibody (96.1.23) (Catalog No. SC-56076), Mfn1 antibody (D-10) (Catalog No. Sc-166644) and Mfn2 antibody (XX-1) (Catalog No. sc-100560) from Santa Co. Ltd.; Nrf2 polyclonal antibody (Catalog No. 16396-1-AP) from Proteintech Co. Ltd.; Drp1 antibody (recombinant Anti-DRP1 antibody) (Catalog No. Ab184248) from Abcam Co. Ltd.,; beta actin Polyclonal Antibody (Catalog No. E-AB--20058), Goat Anti-Rabbit IgG (H+L) (Catalog No. E-AB-1003), Goat Anti-Mouse IgG (H+L) (Catalog No. E-AB-1001) from Elabscience Co. Ltd. The Tecan Safire2 full-wavelength multifunctional microplate reader was purchased from Tecan Group Co. Ltd., Switzerland, and the FUSION FX7 multifunctional imaging system was purchased from Vilber Co. Ltd., France.

### The establishment of cognitive function of rats for the water maze

The training of rats’ cognitive abilities was carried out on the fixed platform of the water maze before operation. The water maze was divided into four quadrants, and the platform was fixed in the fourth quadrant. From the first day of administration, one quadrant (not repeated) was selected every day during the learning period (the first four days), and the rats were positioned in the quadrant in a fixed order (Sorted by groups: Group A 1-6,Group B 7-12,Group C 13-18,Group D 19-24,Group E 25-30).

The memory time of each rat was 50 seconds, and finding the platform marked successful training (if the rats were not able to locate the platform within 50 seconds, they were directed to the platform and stayed there for 30 seconds, which was also recorded as training). During the learning period, all the rats were trained four times a day in the same quadrant. (All the 30 rats ran once, and then repeated the second time from the rat numbered 1 until the 30 rats ran 4 times, indicating that the water maze in this quadrant ended at this time). After the training of the learning period, the rats were put in the second quadrant from the fifth day, which was farthest from the platform, in a fixed order, and the time from entering the water to swimming to the fixed platform was recorded. It was performed once a day to exercise the cognitive abilities of the rats until the day of operation. On the 1st, 3rd, and 5th day after the operation, the rats were again placed in the second quadrant in order (excluding the rats that had been taken), and the time ratio of the rats from entering the water to swimming to the fixed platform was noted.

### The establishment of rat tibial fracture model and treatment of related hippocampal tissues

Thirty SD rats were assorted randomly into five groups as per the number table method: blank control group (α), solvent group (β), NBP group (γ), agonist group (δ), and inhibitor group (ε), with 6 rats per group. The blank control group rats were administered an intraperitoneal injection with 2 ml of normal saline 5 days, 3 days, and 1 day before operation, respectively. The solvent group rats were administered intraperitoneal injection with 2 ml of PEG300 and normal saline (1:1) 5 days, 3 days, and 1 day before operation, respectively. The rats present in the NBP group were administered an intraperitoneal injection with 2 ml NBP solution (40 mg/kg) (dissolved in a mixture of PEG300 and normal saline at a ratio of 1:1) 5 days, 3 days, and 1 day before operation, respectively. The agonist group rats were administered intraperitoneal injection with 1 ml NBP solution (40 mg/kg) (dissolved in a mixture of PEG300 and normal saline at a ratio of 1:1) and 1 ml TBHQ solution (40 mg/kg) (dissolved in a mixture of PEG300 and normal saline at a ratio of 1:1) 5 days, 3 days and 1 day before operation, respectively. The rats present in the inhibitor group received an intraperitoneal injection with 1 ml NBP solution (40 mg/kg) (dissolved in a mixture of PEG300 and normal saline at a ratio of 1:1) and 1 ml Bru (1 mg/kg) (dissolved in a mixture of PEG300 and normal saline at a ratio of 1:1) 5 days, 3 days and 1 day before operation, respectively. On the day of operation, the rats in the solvent, NBP, agonist, and inhibitor groups were administered an intraperitoneal injection with chloral hydrate and isoflurane inhalation anesthesia to establish the rat tibial fracture model. After the establishment of the model, two rats from each group were killed after operation on the 1^st^, 3^rd,^ and 5^th^ day, and the whole brain of one rat was taken and placed in 4% cell fixative for HE staining and immunohistochemistry. The hippocampus of the other rat was removed for the test of related proteins under the Western blot method and also for transmission electron microscopy.

### Hematoxylin–eosin (HE)

Soon after conducting the behavioral test, two rats were selected at random from all the groups, and pentobarbital sodium (50 mg/kg, intraperitoneal injection) was used to anesthetize them on the 1^st^ day, 3^rd^ day and 5^th^ day after the operation, respectively. They were also rapidly perfused with normal saline and 4% paraformaldehyde, one at a time, via the left ventricle and ascending aorta. After performing perfusion, the entire brains of both rats were immediately taken out and submerged for 48 hours in 4% paraformaldehyde, later embedded in paraffin. Moreover, to conduct HE staining, 5 μm thick slices were cut from the Coronal brain.

### Western blot

After conducting the Morris water maze test, six rats from each group were euthanized under deep anesthesia with pentobarbital sodium (50 mg•kg-1, i.p.). Moreover, the hippocampal tissues were collected from the brain immediately and stored at a low temperature of –80° C. Homogenization of the frozen hippocampal tissue was carried out in Radioimmunoprecipitation assay (Solarbio, Beijing, China) buffer, and incubation was performed for 30 min at 4° C, and the supernatant was isolated. The concentration of protein was assessed by employing the bicinchoninic acid method, and extraction of the nuclear proteins was carried out in accordance with a previous report. Equal amounts of protein (30 μg) from all the samples were separated by employing 10% sodium dodecyl sulfate-polyacrylamide gel electrophoresis, which was transferred to polyvinylidene difluoride membranes. Furthermore, 5% skim milk along with Tris-buffered saline Tween-20 was utilized to block the membrane at room temperature for around 2 hours, and incubation of these proteins was carried out overnight at 4° C with primary antibodies under mild shaking. The following day, incubation of the membranes was conducted by employing horseradish peroxidase conjugates. The secondary antibody (goat anti-rabbit IgG, 1: 10,000; Proteintech, Chicago, IL, USA) was left for about 1-2 hours at room temperature, and the antibodies for β-actin were utilized as a loading control for nuclear and total proteins, respectively. Detection of proteins was carried out by employing the electrochemiluminescence (ECL) method; additionally, scanning of the bands was carried out, and ImageJ software (version 1.30v; Wayne Rasband, National Institutes of Health, USA) was employed for the evaluation of the relative density of all the bands [9].

### Statistical analysis

IBM SPSS Statistics 22 statistical software was employed for analysis. The behavioral data of rats in the water maze was gathered and evaluated by ANY maze software. Bartlett test was used to test the homogeneity of variance for the relative gray value of Nrf 2 protein, and the data was similar to the normal distribution. Analysis of variance was employed to conduct the comparison of mean values among multiple groups, and the LSD-t test was employed to perform the pairwise comparison of different groups. When P < 0.05, the variation was considered statistically significant.

## RESULTS

### Result of the water maze test

### Location navigation test

**Figure 1:**
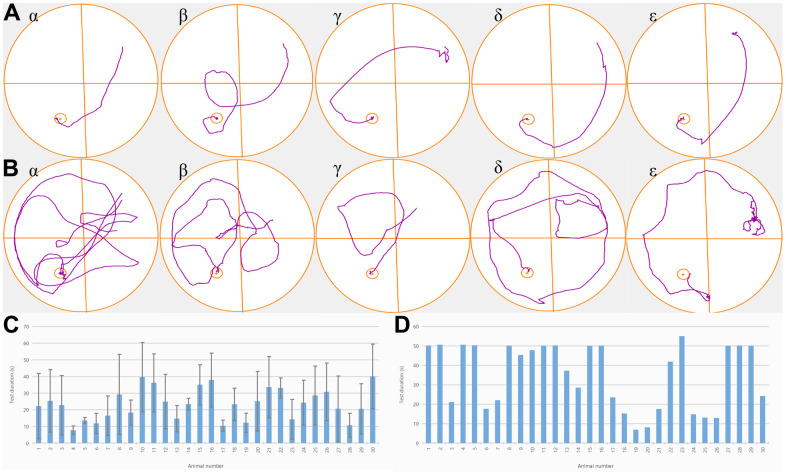
**NBP treatment improved the behavioral deficits of rats with POCD.** (**A**) Trajectory plots of one randomly selected rat from each of the five groups. One day before the operation, all rats could find a fixed platform within 50 seconds, and there was no substantial variation between the groups. (**B**) Trajectory plots of one rat were randomly selected from each of the five groups one day after the operation. (**C**) After the fracture model was established, we observed that a significantly longer time was required to reach the platform by rats in groups α and β in comparison to the ones in groups γ, δ, and ε. (**D**). Time for each rat to reach the platform after the operation.

The training progressively reduced every rat’s escape latency; especially, the escape latency of rats in the NBP, agonist, and inhibitor groups was substantially shortened (P<0.01) in comparison to the blank control and solvent groups. There was no significant difference in escape latency among NBP group, agonist group and inhibitor group (P<0.05). Furthermore, the NBP treatment resulted in the alleviation of poor performance and the escape latency was considerably shorter in the NBP treatment group as compared to the solvent group (P<0.01) (Figure 1A, 1B).

### Spatial probe test

There were substantial variations in the proportion of time spent in the target quadrant between the five groups. The rats in the NBP, agonist, and inhibitor groups spent considerably more time in the target quadrant (P<0.01) as compared to the blank control group and the solvent group, and it shortened the time for rats to reach the platform, indicating that NBP treatment significantly improved the cognitive function of rats after surgery (P<0.01) (Figure 1C, 1D).

### NBP attenuated POCD-induced morphological changes in rat hippocampus

**Figure 2:**
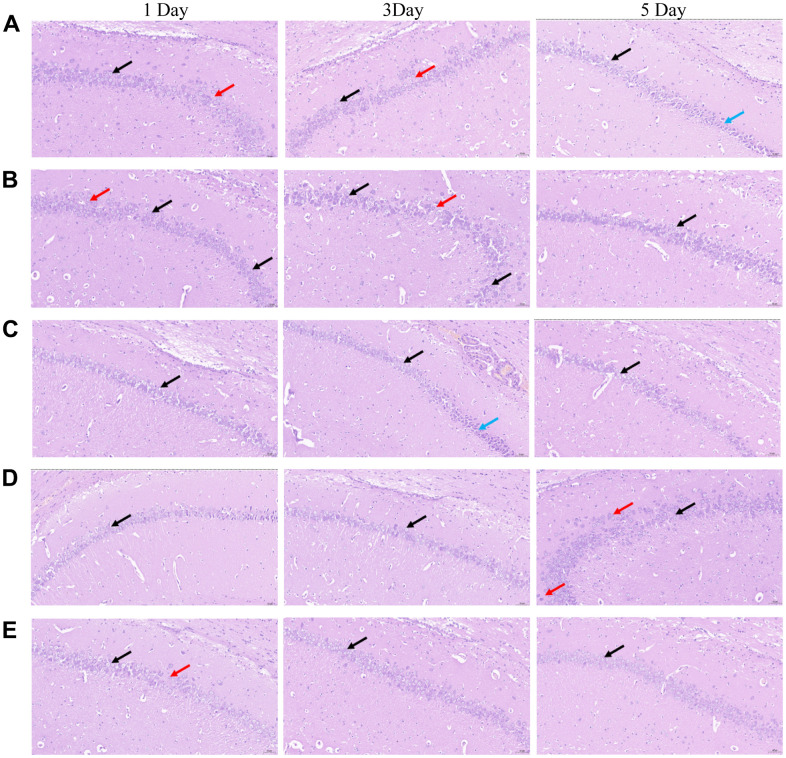
**Morphological changes in the CA 1 region of the hippocampi of rats in the five groups were observed by conducting HE staining.** (**A**) blank group, (**B**) solvent group, (**C**) NBP group, (**D**) NBP + agonist group, and (**E**) NBP + inhibitor group. (Scale bar = 20 μm, magnification = 400 ×).

As shown in Figure 2, the chromatin material of pyramidal cells in CA1, CA2, and CA3 regions of hippocampi in each group was reduced, the nucleus was lightly stained and the cytoplasm was loose (black arrow), but no abnormality was found in the DG region. There was no apparent necrosis and inflammatory cell infiltration. Some pyramidal cells in the CA1 region of the hippocampus (A1, A3, B1, B3, D5, E1) were loose and irregularly arranged (red arrow); In some hippocampi (A5, C3), a small number of pyramidal cells in CA2 and CA3 were contracted, the volume became smaller, the staining was deepened, the basophilia was enhanced, and the cytoplasmic and nuclear boundaries were not clear (blue arrow). After NBP treatment, the morphological changes of group C, D and E were improved compared with group A and B.

### Result of Western blot

**Figure 3:**
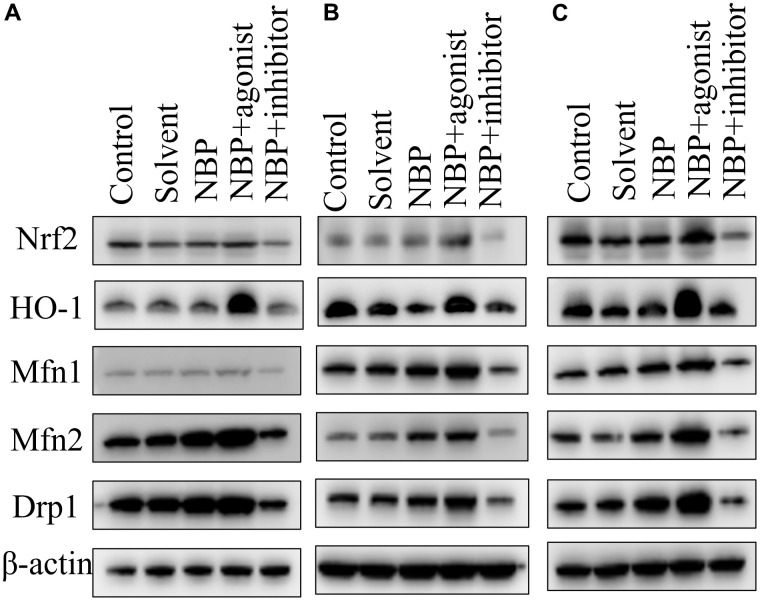
**NBP’s effects on the expressions of Nrf2, Mfn1, Mfn2, and Drp1 in hippocampal tissues by Western blots.** (**A**) one day after the operation. (**B**) three days after the operation. (**C**) five days after the operation.

The grouping of each band (except the marker) was blank control group, solvent group, NBP group, agonist group, and inhibitor group from left to right. Nrf 2, HO-1, Mfn1, Mfn2, Drp1, and β-actin were detected on the 1^st^, 3^rd,^ and 5^th^ day after the operation. The NBP group revealed higher protein expressions of Nrf 2, HO-1, Mfn1, Mfn2, and Drp1 as compared to the blank control and solvent groups. Moreover, substantially higher expression of related proteins was observed in the agonist group in comparison to the NBP group, whereas substantially lower expression of related proteins was detected in the inhibitor group in comparison to the NBP group (Figure 3).

### Immunohistochemical analysis of the expressions of apoptosis-related proteins caspase-3 and caspase-9 in hippocampal tissues

Aipathwell software was used to analyze the area density of caspase-3 and caspase-9 in hippocampal tissues.

Area density = integrated optic density (IOD) / tissue square measure of the area to be tested.

The integrated optic density was the integral of the optical densities of all positive signals (the positive expression of both antibodies was brown), divided by the tissue square measure of the area to be tested, which could reflect the number and depth of positive signals with direct ratio. This method has the advantage that the positive square measure and the positive depth were considered, yet it was not influenced by the size of the tissue to be tested.

**Figure 4:**
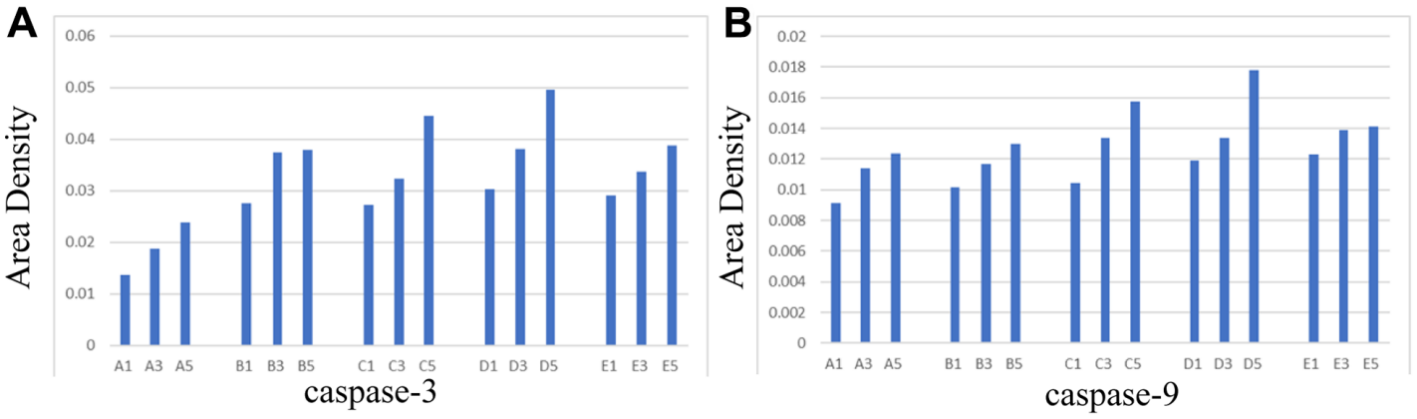
**Area density of hippocampal tissues in each group.** (**A**) caspase-3, (**B**) caspase-9.

It is illustrated in Figure 4 that the area density of antibodies increased with the prolongation of survival time of rats in the same group; the area density of antibodies of rats killed on the same day after the operation was higher in treated groups (Group C, D, E) than in control groups (Group A, B). In the treated group, the growth rate of the antagonist group (Group E) was considerably lower in comparison to the NBP group (Group C) and the agonist group (Group D).

### Morphological changes of mitochondria observed by transmission electron microscopy

The electron micrographs of hippocampal tissues in each group revealed that, as compared to the treated groups (Group C, D, E), the inner and outer mitochondrial membranes in control groups (Group A, B) were significantly damaged- the crista structure was blurred, the neuronal nuclear matrix was missing, the cytoplasmic organelles were blurred, and the number of protrusions was less.

**Figure 5:**
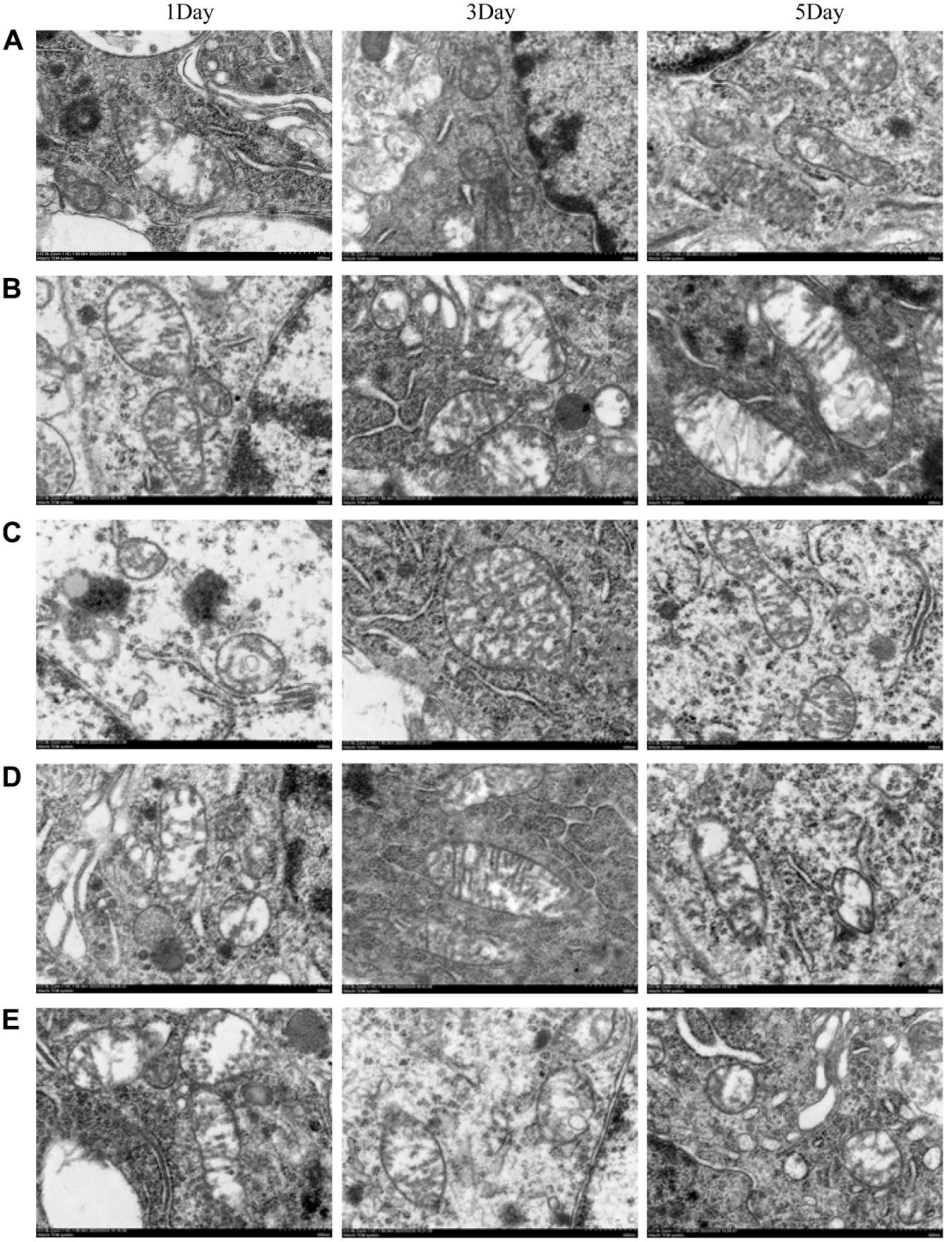
**The morphological changes of mitochondria were observed by transmission electron microscopy.** (**A**) blank group, (**B**) solvent group, (**C**) NBP group, (**D**) NBP + agonist group, and (**E**) NBP + inhibitor group. (Scale bar = 20 μm, magnification = 1200x).

According to the results of transmission electron microscopy, NBP could protect the integrity of mitochondrial membranes and organelles and maintain normal mitochondrial dynamics by activating the Nrf 2/ARE signaling pathway, thus alleviating postoperative cognitive dysfunction (Figure 5).

## DISCUSSION

The pathogenesis of POCD is complex, including protein misfolding/aggregation, amyloid β aggregation, oxidative stress, mitochondrial dysfunction, glial cell dysfunction, excitotoxicity, calcium processing dysfunction, and neuroinflammation [10]. Moreover, oxidative stress and mitochondrial damage are essential links in the pathogenesis of POCD [11]. Furthermore, a considerably large number of research works have revealed that although the causes of neurocognitive disorders are different, there are some similar pathological injury mechanisms in these diseases, such as mitochondrial damage, excessive ROS production, nervous system inflammation, and so on [12, 13]. Studies have confirmed that abnormal mitochondrial dynamics is an early sign of neurocognitive disorders [14], which happens before the amyloid beta protein aggregation, phosphorylation of Tau protein, and neuronal apoptosis. Therefore, if we can find the signaling pathway to improve the mitochondrial dynamic abnormalities, we can intervene in POCD in the earlier stages and avoid its progression from mild to severe cognitive impairment.

NBP is a multi-target drug with multiple protective effects on the brain tissue, and a large number of studies have revealed that NBP can block POCD-induced cognitive function [15, 16]. For instance, NBP develops spatial learning/memory of the Wistar Kyoto rats with spontaneous hypertension by bilateral occlusion surgery, as their performance on the MWM test is substantially enhanced [7, 17]. Furthermore, it is exhibited that the administration of NBP can counteract cognitive impairment as well as plays a vital role in the reversal of the abnormal neuronal morphology in the hippocampus region of rats with chronic cerebral ischemia detected by HE [16].

The Nrf 2/ARE signaling pathway is a classical anti-oxidative stress pathway, which is regulated by nuclear factor E2 related factor 2 (Nrf 2) [18, 19]. Under physiological conditions, the binding of Nrf 2 to KEAP1 is localized in the cytoplasm, and Nrf 2 is regulated at low protein levels [20]. Under oxidative stress, when Nrf 2 is activated, its dissociation from KEAP1 takes place, and it moves inside the nucleus and binds to the antioxidant response element (ARE) to initiate the Nrf 2/ARE signaling pathway [21]. Nrf 2 induces and expresses genes regulated by EpRE after activation. The target genes of Nrf 2 include glutathione (GSH)-regulating enzymes, antioxidant proteins/enzymes, drug-metabolizing enzymes or transporters regulated by drugs, proteasome subunits, pentose phosphate pathway enzymes, and enzymes involved in nucleotide synthesis. Therefore, Nrf 2/ARE signaling pathway plays a significant function in the cellular defense against oxidative stress. Moreover, Nrf 2 also enhances autophagy via p62/SQSTM1 and there is increasing evidence that activation of the Nrf 2-mediated antioxidant pathway can play a protective role in a variety of neurodegenerative diseases [22–24]. Recently, the novel function of Nrf 2 has been found to affect the regulation of mitochondrial membrane potential and the synthesis of ATP substrates in the respiratory chain. In addition, some studies have found that the lack of Nrf 2 molecules in isolated mitochondria can lead to mitochondrial fatty acid peroxidation and eventually lead to energy production disorders. Some other studies have also shown that the activation of the Nrf 2 pathway promotes mitochondrial dynamic fusion activity and inhibits mitotic activity [25] and it can also affect mitochondrial autophagy by regulating PINK expression [26]. The function of the Nrf 2 pathway in the regulation of mitochondrial function is becoming an area of interest for the scientific fraternity; moreover, it is considered to be a critical regulator that links mitochondrial function to oxidative stress, making itself a potential therapeutic target for studies of Neuroprotection.

This research found that the occurrence of postoperative cognitive dysfunction in treated groups (NBP, and Nrf 2 agonist groups) was substantially lower in comparison to the blank control and solvent groups (P < 0.05). The Western blot findings revealed higher protein expressions of Nrf 2, HO-1, Mfn1, Mfn2, and Drp1 in the NBP group in comparison to the blank control and solvent groups. The expression of related proteins in the agonist group was substantially upregulated in comparison to the NBP group, and the expression of related proteins in the inhibitor group was substantially downregulated in comparison to the NBP group. These results suggested that the consequence of NBP on postoperative cognitive function of rats after surgery is controlled by Nrf 2 pathway. In this study, we found that NBP could increase the expressions of HO-1 and Nrf 2 in a dose-dependent manner, indicating that the antioxidant effect of NBP in rats with tibial fracture might be achieved by overexpression of the Nrf 2/ARE pathway. In summary, we observed that NBP could affect the cognitive function of rats after surgery by turning on the Nrf 2/ARE signaling pathway.
